# Urban farming in Hong Kong

**DOI:** 10.1186/2193-1801-4-S2-P9

**Published:** 2015-11-27

**Authors:** Daniel Wai-tin Chan

**Affiliations:** Faculty of Science and Technology, Technological and Higher Education Institute of Hong Kong, Hong Kong

## Background

Suitable indoor green plants have been tested and shown to reduce Volatile Organic Compounds by 80% and carbon dioxide by up to 25% in singe offices, offering significant health benefits to office workers [1, 2].

Further studies have also shown that a leafy office relieves stress and reduces negative mood states by up to 60% and just one plant is enough to make a positive difference in the workplace. This research has shown that plants in an office environment can result in 37% reduction in tension/anxiety, a 58% reduction in depression/dejection, a 44% reduction in anger/hostility, a 38% reduction in fatigue, a 30% reduction in confusion and a 5% increase in vigour [3–6].

Other researchers advocate growing green vegetables indoor which grow faster in higher carbon dioxide concentration indoor, and faster than outside in winter since the indoor space in warm. Green vegetables turn carbon dioxide into food eaten by people in the food chain, i.e. as fuel and food for humans, contributing significantly to absorbing carbon dioxide than growing non-food green plants which soon will mostly decay and turn into atmospheric carbon dioxide again [7, 8].

## Results

This paper attempts to integrate all the above benefits of growing indoor green plants together by proposing an illustrative selection of a variety of green plants which will demonstrate collectively the above benefits inside an office.

Furthermore the more carbon dioxide, which is emitted by humans indoor, absorbed into indoor green plants, which also enhance indoor air quality, the more energy is saved in heating up or cooling down outside air which is needed traditionally to dilute indoor air, by exhausting the same amount to maintain an acceptable level of indoor air quality, because the outdoor air intake will be much reduced. This will offer extra benefits to cities such as Beijing which is suffering from dust storms, demanding expensive and energy in filtering outside air for intake into buildings.

## Conclusions

The overall benefits as discussed will expectedly bring a new paradigm in interior space utilization in greening and growing vegetables and other green plants in the built environment. This will also contribute to combating climate change.
